# Ferroptosis in autoimmune uveitis: potential links to blood–retinal barrier dysfunction and retinal inflammation

**DOI:** 10.3389/fimmu.2026.1907512

**Published:** 2026-09-02

**Authors:** Jun Wang, Heping Li, Haohan Xiong, Jia He, Ting Que, Xing Fang

**Affiliations:** Department of Ophthalmology, Guangyuan Central Hospital, Guangyuan, Sichuan, China

**Keywords:** autoimmune uveitis, blood-retinal barrier, ferroptosis, myeloid cells, oxidative stress, retinal pigment epithelium, retinal vascular endothelium

## Abstract

Autoimmune uveitis (AU) is a vision-threatening intraocular inflammatory disease characterized by disruption of ocular immune privilege, blood-retinal barrier (BRB) dysfunction, and progressive immune-mediated retinal injury. Although corticosteroids and conventional immunosuppressive therapies remain clinically effective, their long-term use is limited by adverse effects, relapse, and incomplete disease control. Ferroptosis is an iron-dependent form of regulated cell death driven by phospholipid peroxidation. It may provide a mechanistic link between oxidative stress, retinal cell injury, and inflammatory amplification. The retina is potentially susceptible because of its high oxygen demand, redox-active iron, and enrichment in polyunsaturated fatty acids. Direct evidence in AU, however, remains limited. In experimental autoimmune uveitis (EAU), fine particulate matter with an aerodynamic diameter of 2.5 μm or less (PM_2.5_) induced ferroptosis-related changes in CD4^+^ T cells and enhanced T helper 17 (Th17) pathogenicity, whereas modulation of transforming growth factor-β receptor 1 (TGFBR1) altered the glutathione peroxidase 4 (GPX4)/cystine–glutamate antiporter (xCT) axis, lipid peroxidation, and inflammatory severity. Studies in retinal pigment epithelial cells, retinal vascular endothelial cells, and other retinal injury models provide supporting evidence but should not be interpreted as direct proof in AU. This Mini Review examines ferroptosis as a potential contributor to BRB dysfunction and retinal inflammation. It separates direct AU/EAU findings from extrapolated evidence and highlights priorities for preclinical validation.

## 1 Introduction

Autoimmune uveitis (AU) is a vision-threatening noninfectious intraocular inflammatory disorder in which loss of tolerance to ocular antigens and dysregulated adaptive immunity contribute to tissue injury (1–4). Uveitis is anatomically classified as anterior, intermediate, posterior, or panuveitis according to the principal site of inflammation (1). Because the present review focuses on the blood-retinal barrier (BRB), its discussion is most directly applicable to intermediate, posterior, and panuveitis and to experimental autoimmune uveitis (EAU), a widely used model of retinal autoimmunity. Although glucocorticoids and conventional immunosuppressive regimens remain effective, long-term treatment is limited by adverse effects, relapse, and incomplete disease control.

Ferroptosis is a nonapoptotic form of regulated cell death caused by iron-dependent accumulation of oxidized phospholipids (5, 6). It occurs when lipid hydroperoxide generation exceeds cellular defense capacity. The cystine/glutamate antiporter system Xc^-^/glutathione (GSH)/glutathione peroxidase 4 (GPX4) axis is the best-characterized defense, while parallel pathways involving ferroptosis suppressor protein 1 (FSP1), dihydroorotate dehydrogenase (DHODH), and GTP cyclohydrolase 1 (GCH1) provide additional protection (7–9). The retina may be particularly susceptible to ferroptotic stress because of its high oxygen consumption, continuous light exposure, redox-active iron, and polyunsaturated fatty acid (PUFA)-rich membranes (10, 11).

In a fine particulate matter with an aerodynamic diameter of 2.5 μm or less (PM_2.5_)-exposed EAU model, ferroptosis-related changes in CD4^+^ T cells accompanied enhanced T helper 17 pathogenicity, and ferrostatin-1 attenuated the exposure-associated worsening of inflammation (12). These findings identify PM_2.5_ as an experimental disease modifier rather than a universal initiating trigger. A separate study identified transforming growth factor-β receptor 1 (*TGFBR1*) as a candidate ferroptosis-related gene and showed that its modulation altered retinal lipid peroxidation and the GPX4/xCT defense axis, together with inflammatory severity in EAU (13). Recent reviews have summarized regulated cell-death pathways in uveitis and ferroptosis across ocular diseases (14, 15). Building on these broader reviews, this Mini Review focuses specifically on whether ferroptotic stress may contribute to BRB dysfunction and retinal inflammation in AU, while clearly separating direct EAU evidence from findings in other ocular and non-ocular models.

## 2 Role of the BRB in autoimmune uveitis

The BRB is a specialized anatomical and functional interface that preserves retinal homeostasis and immune privilege. It comprises anatomically distinct inner and outer components. The inner BRB is formed by tight junctions between retinal vascular endothelial cells and is supported by pericytes, astrocytes, Müller glia, and the vascular basement membrane. The outer BRB is formed by tight junctions between adjacent retinal pigment epithelial (RPE) cells and regulates exchange between the choroid and neural retina (16). The neural retina lies between these two barriers, while the fenestrated choriocapillaris is separated from it by the RPE and Bruch’s membrane.

In EAU, leukocyte adhesion and trafficking are temporally associated with BRB leakage and increased endothelial adhesion-molecule expression (17). Leukocyte diapedesis can produce transient, localized loss or redistribution of endothelial tight-junction proteins (18). In cultured human retinal endothelial cells, TNF-α and IL-1β reduce barrier resistance and alter junctional organization (19). The outer BRB is also active in disease: CUB domain-containing protein 1 (CDCP1)-dependent regulation of RPE junctional integrity changes lymphocyte migration across the RPE and influences EAU severity (20). Single-cell analyses further show inflammation-associated changes in photoreceptors, Müller glia, RPE cells, and retinal myeloid populations (21).

**Figure 1:**
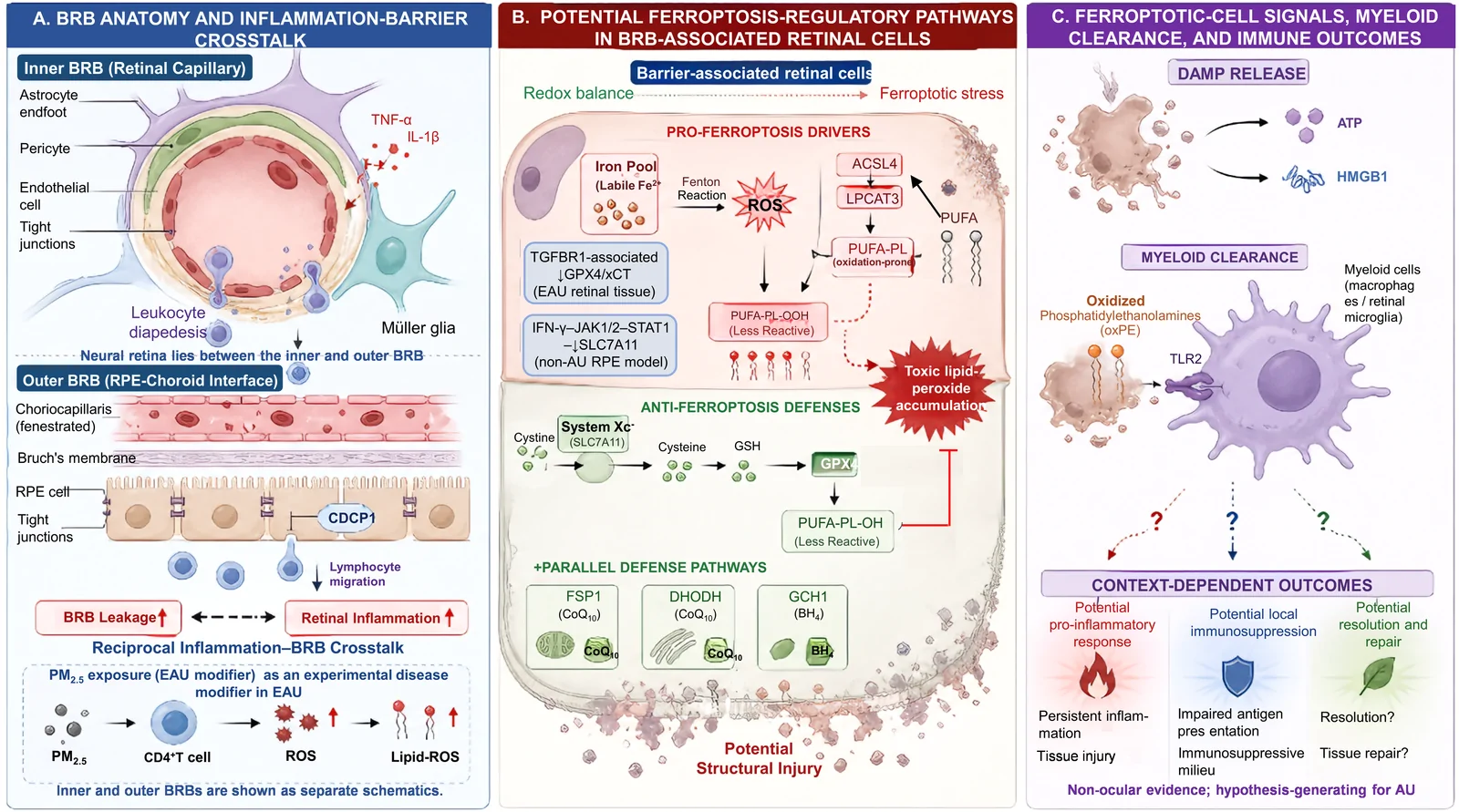
BRB anatomy, ferroptosis-regulatory pathways, and immune crosstalk in autoimmune uveitis. **(A)** Anatomically distinct inner and outer blood–retinal barriers (BRBs) and reciprocal crosstalk between BRB leakage and retinal inflammation. PM2.5 is shown as an experimental disease modifier in EAU rather than a universal trigger. The inner and outer BRBs are shown as separate schematics; *in vivo*, the neural retina lies between them. **(B)** Major pro-ferroptotic and anti-ferroptotic pathways relevant to BRB-associated retinal cells. The TGFBR1-associated GPX4/xCT phenotype derives from EAU retinal tissue, whereas the IFN-γ–JAK1/2–STAT1–SLC7A11 pathway represents supporting evidence from a non-AU RPE model. **(C)** Ferroptotic-cell signals, myeloid clearance, and context-dependent immune outcomes. The illustrated pro-inflammatory, immunosuppressive, and resolution/repair outcomes are hypothesis-generating extrapolations from non-ocular studies rather than established mechanisms in AU.

BRB dysfunction and retinal inflammation likely reinforce each other rather than occurring in a fixed order. Cytokines, leukocyte diapedesis, and VEGF-mediated transcytosis can increase permeability without requiring death of barrier-forming cells (18, 19, 22). These functional changes may be reversible. Completed ferroptosis, in contrast, may entail irreversible loss of affected cells and could convert functional barrier dysregulation into structural injury. Ferroptosis is considered here a potential contributor to BRB dysfunction rather than its sole cause. **Figure 1** integrates BRB anatomy and reciprocal inflammation–barrier crosstalk with ferroptosis-regulatory pathways in BRB-associated retinal cells and the potential immune consequences of ferroptotic-cell clearance.

## 3 Ferroptosis regulation relevant to BRB-associated retinal cells

Ferroptosis is initiated when oxidizable phospholipids accumulate and are converted to lipid hydroperoxides in an iron-dependent metabolic setting (5, 6). Relevant sources of reactive oxygen species (ROS) include mitochondrial electron leakage, NADPH oxidases, iron-catalyzed Fenton chemistry, inflammatory-cell respiratory bursts, and retinal photo-oxidative reactions (10, 23). ROS accumulation alone does not define ferroptosis. The decisive event is uncontrolled phospholipid peroxidation in the presence of permissive iron availability and insufficient lipid peroxide-repair capacity. **Table 1** summarizes the direct EAU evidence and the supporting evidence from other ocular models.

**Table 1:** Evidence framework linking BRB dysfunction, retinal inflammation, and ferroptosis-related mechanisms in autoimmune uveitis.

Mechanism or context	Principal cells/model	Main observation	Evidence relevance to AU	Interpretive limit
Junctional remodeling and transcytosis	Retinal vascular endothelium; EAU and endothelial models	Cytokines, leukocyte diapedesis, and VEGF-associated transcytosis increase BRB permeability without requiring barrier-cell death (18, 19, 22).	Direct EAU and ocular endothelial evidence for functional BRB dysfunction.	Supports barrier dysregulation, but not ferroptosis-specific injury.
Leukocyte trafficking and BRB dysfunction	EAU; retinal vascular endothelium	Leukocyte adhesion and diapedesis accompany BRB leakage and transient junctional disruption (17, 18).	Direct EAU evidence for reciprocal inflammation-barrier injury.	Does not establish ferroptotic death as the cause of BRB dysfunction.
RPE barrier regulation	EAU; RPE/outer BRB	CDCP1-dependent regulation of RPE junctional integrity alters lymphocyte migration across the RPE and influences EAU severity (20).	Direct EAU evidence for outer-BRB involvement.	Shows outer-BRB relevance; RPE ferroptosis itself is untested.
PM_2.5_-exposed EAU	CD4^+^ T cells; retina	PM_2.5_ exposure alters Fe^2+^, ROS, lipid ROS, MDA, glutathione balance, and ferroptosis-related proteins; Th17 pathogenicity and EAU severity increase, and ferrostatin-1 partly attenuates the exposure-associated phenotype (12).	Direct AU/EAU evidence for a ferroptosis-associated immune phenotype in a defined exposure-modified model.	Cannot confirm PM_2.5_ as a universal AU trigger or ferroptosis across all retinal cell types.
TGFBR1-related EAU	Retina; immune phenotype	TGFBR1 upregulation reduces the GPX4/xCT defense axis, increases retinal MDA, and aggravates clinical and histopathological inflammation; pharmacological inhibition reverses these changes (13).	Direct AU/EAU evidence for a TGFBR1-associated redox-immune axis.	Cell-specific ferroptotic death and the primary target cell population remain to be demonstrated.
Cultured RPE ferroptosis	RPE/outer BRB	GSH depletion induces lipid peroxidation, ferroptosis-associated injury, autophagy, and premature senescence in RPE cells (27).	Supports the susceptibility of RPE cells to ferroptotic stress.	*In vitro* redox-stress model; not direct evidence in AU.
IFN-γ-associated RPE ferroptosis	AMD-related inflammatory RPE model	IFN-γ promotes RPE ferroptosis through JAK1/2-STAT1-dependent suppression of *SLC7A11*/xCT (28).	Provides an ocular cytokine-to-ferroptosis link in RPE cells.	Disease context differs from AU, so its relevance is supportive rather than direct.
PM_2.5_ retinal endothelial injury	Retinal vascular endothelium/inner BRB	PM_2.5_ induces inflammation-associated ferroptotic injury and impairs inner-BRB integrity in retinal vascular endothelial cells (29).	Supports endothelial ferroptotic vulnerability in an ocular barrier context.	Exposure model is not autoimmune uveitis.
Retinal ischemia-reperfusion and glaucoma models	Retinal neuronal, glial, and vascular populations, including RGCs	Ferroptosis-related signatures, iron dysregulation, and ferroptosis-responsive injury have been reported in defined retinal injury models (30, 31).	Demonstrates cell-type susceptibility to ferroptotic injury in retinal disease.	Cannot be directly generalized to AU or BRB pathology.
Ferroptotic-cell signals and myeloid responses	Non-ocular tumor and invertebrate models; ferroptotic cells and phagocytes	ATP/HMGB1 release, surface oxidized phospholipids, TLR2-linked phagocytosis, and divergent immune outcomes have been reported (39–43).	Provides a mechanistic framework for considering ferroptotic-cell clearance and myeloid responses.	Derived from non-ocular systems; AU-specific relevance remains unresolved.

This table separates direct AU/EAU evidence from ocular and non-ocular extrapolations. Supporting evidence should not be interpreted as direct proof in AU. Abbreviations are defined in the main abbreviation list.

The system Xc^-^/GSH/GPX4 axis limits phospholipid hydroperoxide accumulation: system Xc^-^ imports cystine for GSH synthesis, and GPX4 uses GSH to reduce phospholipid hydroperoxides to less reactive alcohols (5, 24). Parallel defenses include FSP1-coenzyme Q_10_, mitochondrial DHODH-coenzyme Q_10_, and GCH1-derived tetrahydrobiopterin pathways (7–9). Iron uptake, storage, export, and ferritinophagy regulate the labile iron pool and therefore influence ferroptotic susceptibility (6, 23, 25). These pathways provide a mechanistic framework, but their direct contribution to AU must be evaluated in the relevant retinal and immune cell types. These core pro- and anti-ferroptotic pathways are summarized in **Figure 1B**.

### 3.1 Ferroptosis-related metabolism and BRB-associated retinal vulnerability

Iron homeostasis and phospholipid composition are major determinants of ferroptotic sensitivity. Labile Fe^2+^ promotes Fenton chemistry, whereas PUFAs incorporated into membrane phospholipids provide oxidation-prone substrates (5, 6, 23). Acyl-CoA synthetase long-chain family member 4 (ACSL4)-dependent PUFA activation and lysophosphatidylcholine acyltransferase 3 (LPCAT3)-mediated phospholipid remodeling expand the pool of oxidation-prone PUFA-containing phospholipids (PUFA-PLs) (26). Because PUFAs are also essential for photoreceptor membrane properties and visual signaling, their peroxidation may impair retinal function as well as promote cell death.

Evidence differs among retinal cell types. In cultured RPE cells, GSH depletion induces lipid peroxidation and ferroptosis-associated injury (27). In an age-related macular degeneration-related model, interferon-γ (IFN-γ) promoted RPE ferroptosis through JAK1/2-STAT1-dependent suppression of *SLC7A11*, which encodes xCT (28). In a non-AU retinal endothelial model, PM_2.5_ has also been reported to damage retinal vascular endothelial cells and the inner BRB through inflammation-associated ferroptotic mechanisms (29). Retinal ischemia-reperfusion and glaucoma models provide additional evidence in retinal neuronal, glial, and vascular populations, including retinal ganglion cells (30, 31). These studies demonstrate retinal ferroptotic vulnerability under defined stresses without confirming the same process occurs in AU.

The two EAU studies discussed here provide more direct support, though from a narrow set of models. PM_2.5_ exposure altered Fe^2+^, ROS, lipid ROS, malondialdehyde (MDA), glutathione balance, and ferroptosis-related proteins in CD4^+^ T cells and increased EAU severity; ferrostatin-1 alleviated the exposure-associated inflammatory phenotype (12). *TGFBR1* upregulation reduced the GPX4/xCT defense axis, increased retinal MDA, and aggravated clinical and histopathological inflammation, whereas pharmacological inhibition reversed these changes (13). These findings point to a ferroptosis-associated redox phenotype in specific EAU settings, without implying widespread ferroptotic death across every stage or cell type. These direct EAU observations are represented in **Figure 1A** as PM_2.5_-exposed CD4^+^ T-cell evidence and in **Figure 1B** as a TGFBR1-associated retinal redox phenotype.

### 3.2 Signaling pathways linking inflammation, ferroptotic susceptibility, and BRB dysfunction

Inflammatory and cellular stress pathways can modify ferroptotic susceptibility. Relevant pathways include NF-κB, MAPK, JAK-STAT signaling, endoplasmic reticulum stress, autophagy, iron handling, and the system Xc^-^/GSH/GPX4 axis (6, 13, 28, 32). Their effects depend on cell type, stimulus, timing, and metabolic state. NF-κB and MAPK signaling can influence ROS production, cytokine expression, and cell-survival programs, but direct evidence that NF-κB/MAPK pathways themselves suppress xCT or drive ferroptotic BRB dysfunction in AU is lacking (32). One plausible reading is that inflammatory signaling creates a pro-oxidant environment that lowers the threshold for ferroptosis, though this remains to be tested directly.

Two pathway-specific links are supported experimentally in ocular or EAU-related settings. In an age-related macular degeneration-related model, IFN-γ promoted RPE ferroptosis through JAK1/2-STAT1-dependent suppression of *SLC7A11* (28). In EAU, *TGFBR1* modulation altered the GPX4/xCT defense axis and was accompanied by changes in regulatory and T helper 17 (Th17)-associated inflammatory markers (13). Endoplasmic reticulum stress and autophagy also interact with ferroptotic vulnerability. Oxidative stress induces endoplasmic reticulum stress in RPE cells (33), and ferroptotic stress activates related programs in other cell systems (34). Basal autophagy supports immune and tissue homeostasis (35), whereas macrophage-specific disruption of autophagy promotes inflammatory eye disease and can aggravate experimental uveitis (36). Selective autophagic processes such as ferritin degradation can also expand the labile iron pool (25). Thus, autophagy may protect or sensitize cells depending on the process and disease stage.

### 3.3 Ferroptotic-cell signals, myeloid clearance, and context-dependent immune outcomes

Macrophages and retinal microglia occupy heterogeneous and dynamic activation states. The traditional M1/M2 dichotomy is increasingly considered insufficient to capture the mixed, intermediate, and context-dependent myeloid phenotypes revealed by single-cell studies. In EAU, interventions can alter macrophage-associated inflammatory and regulatory programs, while single-cell analyses identify distinct inflammatory microglial states (21, 37, 38). Terms such as pro-inflammatory, regulatory, reparative, or mixed myeloid states are therefore preferable unless a phenotype has been experimentally defined.

Viable activated immune cells and cells undergoing ferroptosis can influence the retina through distinct, partly overlapping routes. Activated myeloid cells and infiltrating lymphocytes contribute to an inflammatory milieu that can impair BRB-associated cells (17–21). As membrane integrity is lost, cells undergoing ferroptosis can release damage-associated molecular patterns (DAMPs), including ATP and high-mobility group box 1 (HMGB1), a prototypical alarmin. Early ferroptotic cancer cells have induced immunogenic responses in one setting, whereas ferroptotic tumor cells impaired dendritic-cell maturation and antigen presentation in another (39, 40). These contrasting observations indicate that immune outcomes may depend on the stage of cell death, tissue context, and responding immune-cell population.

The clearance phase provides another route through which ferroptotic cells could influence inflammation. Oxidized phosphatidylethanolamines can serve as recognition signals for Toll-like receptor 2 (TLR2)-dependent phagocytosis (41). Ferroptotic cell death has also been associated with an immunosuppressive tumor environment (42), whereas ferroptosis-like death prolonged inflammation in Drosophila (43). Because these findings derive from non-ocular systems, they should be viewed as hypothesis-generating for AU rather than as disease-specific evidence. These hypothesis-generating clearance and immune-outcome relationships are summarized in **Figure 1C**. Future AU studies will need to determine whether ferroptotic RPE, endothelial, neural retinal, or immune cells undergo distinct clearance programs, and whether such clearance favors resolution, persistent inflammation, or local immune suppression.

## 4 Preclinical landscape and translational priorities

No ferroptosis-targeted therapy has been established clinically for AU. The limited direct EAU evidence comprises ferrostatin-1 treatment in a PM_2.5_-exposure model and TGFBR1 inhibition in a separate EAU model (12, 13). Mechanistic and ocular studies suggest several potential approaches, including iron chelation, suppression of lipid peroxidation, and preservation of GPX4-dependent or GPX4-independent antioxidant defenses (7–9, 27–31). Modulating inflammatory signals linked to *SLC7A11*/xCT suppression is a further possibility (28). These approaches are preclinical hypotheses for AU, not established therapies.

**Figure 2:**
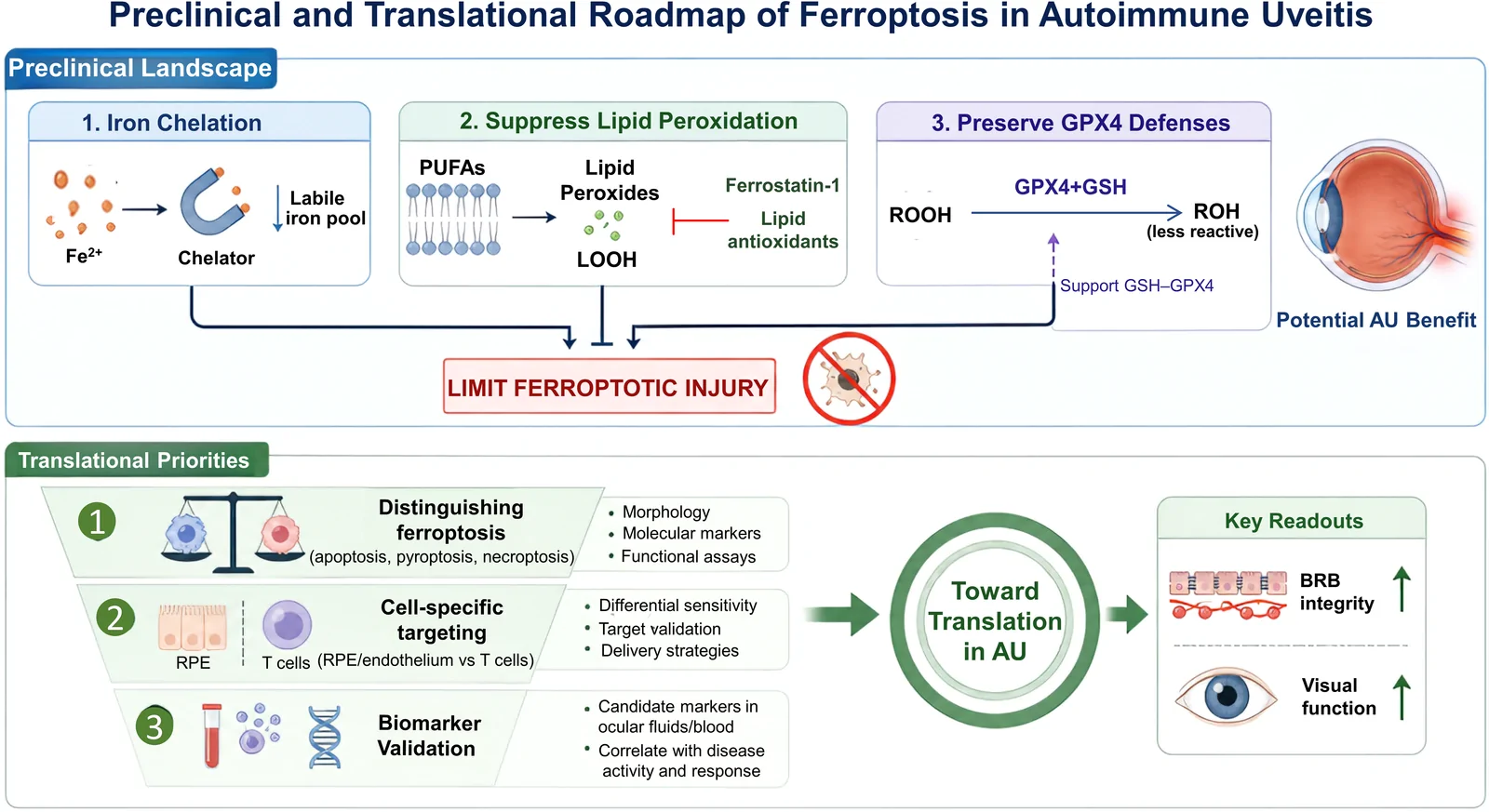
Preclinical and translational roadmap of ferroptosis in autoimmune uveitis. Candidate strategies and key translational priorities are summarized, with BRB integrity and visual function as proposed readouts. All strategies remain preclinical in AU.

Before clinical translation, ferroptosis should be distinguished from apoptosis, necroptosis, pyroptosis, and secondary oxidative injury using several complementary criteria rather than a single marker. The relevant cell type and disease stage must also be identified, because protecting RPE or endothelial cells may have different consequences from altering pathogenic T cells or myeloid populations. No biomarker has been validated as a specific measure of retinal ferroptosis in patients. Near-term studies should test whether ferroptosis inhibitors preserve inner and outer BRB function in EAU. Useful cell-resolved readouts include junctional integrity, vascular leakage, and lipid peroxidation, alongside immune-cell composition and visual function. **Figure 2** summarizes these candidate preclinical strategies and the key validation steps required for translation, including discrimination of ferroptosis from other cell-death programs, cell-specific targeting, biomarker validation, and assessment of BRB integrity and visual function.

## 5 Conclusion

Autoimmune uveitis is driven by interactions among immune activation, oxidative stress, metabolic imbalance, and BRB dysfunction. Ferroptosis offers a plausible mechanistic thread running through these processes — iron dysregulation and phospholipid peroxidation compounding an already impaired antioxidant defense to cause retinal injury. The strongest direct support comes from specific EAU studies, whereas mechanistic work in RPE, retinal endothelial, neural retinal, and immune cells in other ocular models provides supporting evidence. Taken together, the available evidence supports a limited, context-sensitive role for ferroptotic stress in BRB dysfunction and retinal inflammation, without placing ferroptosis as a universal upstream driver of AU.

Future work should map ferroptotic signatures across individual retinal and immune cell types and clarify how ferroptotic cells are cleared in the immune-privileged retina. A key open question is whether targeted inhibition can preserve barrier and visual function without impairing protective immunity. Future studies should preserve this evidentiary separation while testing the proposed mechanisms in cell-resolved AU models.

## References

[1] Jabs DA Nussenblatt RB Rosenbaum JT Standardization of Uveitis Nomenclature (SUN) Working Group . Standardization of uveitis nomenclature for reporting clinical data. Results of the first international workshop. Am J Ophthalmol. (2005) 140:509–16. doi: 10.1016/j.ajo.2005.03.057 16196117 PMC8935739

[2] Caspi RR . A look at autoimmunity and inflammation in the eye. J Clin Invest. (2010) 120:3073–83. doi: 10.1172/JCI42440 20811163 PMC2929721

[3] Horai R Caspi RR . Cytokines in autoimmune uveitis. J Interferon Cytokine Res. (2011) 31:733–44. doi: 10.1089/jir.2011.0042 21787221 PMC3189550

[4] Caspi RR . Understanding autoimmune uveitis through animal models: the Friedenwald lecture. Invest Ophthalmol Vis Sci. (2011) 52:1872–9. doi: 10.1167/iovs.10-6909 21450922 PMC3101683

[5] Stockwell BR Friedmann Angeli JP Bayir H Bush AI Conrad M Dixon SJ . Ferroptosis: a regulated cell death nexus linking metabolism, redox biology, and disease. Cell. (2017) 171:273–85. doi: 10.1016/j.cell.2017.09.021 28985560 PMC5685180

[6] Stockwell BR . Ferroptosis turns 10: emerging mechanisms, physiological functions, and therapeutic applications. Cell. (2022) 185:2401–21. doi: 10.1016/j.cell.2022.06.003 35803244 PMC9273022

[7] Doll S Freitas FP Shah R Aldrovandi M da Silva MC Ingold I . FSP1 is a glutathione-independent ferroptosis suppressor. Nature. (2019) 575:693–8. doi: 10.1038/s41586-019-1707-0 31634899

[8] Mao C Liu X Zhang Y Lei G Yan Y Lee H . DHODH-mediated ferroptosis defence is a targetable vulnerability in cancer. Nature. (2021) 593:586–90. doi: 10.1038/s41586-021-03539-7 33981038 PMC8895686

[9] Kraft VAN Bezjian CT Pfeiffer S Ringelstetter L Müller C Zandkarimi F . GTP cyclohydrolase 1/tetrahydrobiopterin counteract ferroptosis through lipid remodeling. ACS Cent Sci. (2020) 6:41–53. doi: 10.1021/acscentsci.9b01063 31989025 PMC6978838

[10] Böhm EW Buonfiglio F Voigt AM Bachmann P Safi T Pfeiffer N . Oxidative stress in the eye and its role in the pathophysiology of ocular diseases. Redox Biol. (2023) 68:102967. doi: 10.1016/j.redox.2023.102967 38006824 PMC10701459

[11] Tisi A Feligioni M Passacantando M Ciancaglini M Maccarone R . The impact of oxidative stress on blood-retinal barrier physiology in age-related macular degeneration. Cells. (2021) 10:64. doi: 10.3390/cells10010064 33406612 PMC7823525

[12] Liu Y Zhang W Wang H Liu H Yu Q Luo X . Fine particulate matter potentiates Th17-cell pathogenicity in experimental autoimmune uveitis via ferroptosis. Ecotoxicol Environ Saf. (2024) 284:116979. doi: 10.1016/j.ecoenv.2024.116979 39232294

[13] Zhang D Ren H Xing Y Chen Z . Exploring the mechanisms of the ferroptosis-related gene TGFBR1 in autoimmune uveitis based on machine learning models. ACS Omega. (2025) 10:27830–46. doi: 10.1021/acsomega.5c00595 40657117 PMC12242657

[14] Liu J Mao L Chen G Zou L Shi Y Jiang L . Cell death in uveitis: pyroptosis, ferroptosis, apoptosis, and beyond. Front Immunol. (2026) 17:1751523. doi: 10.3389/fimmu.2026.1751523 42164490 PMC13183624

[15] Zhu G Lin Y Han Z Cao H Zhu K Shi R . Ferroptosis and the eye: bridging the gap between cell death and vision preservation. Front Immunol. (2026) 17:1791087. doi: 10.3389/fimmu.2026.1791087 41988183 PMC13076123

[16] Cunha-Vaz J Bernardes R Lobo C . Blood-retinal barrier. Eur J Ophthalmol. (2011) 21:S3–9. doi: 10.5301/EJO.2010.6049 23264323

[17] Xu H Forrester JV Liversidge J Crane IJ . Leukocyte trafficking in experimental autoimmune uveitis: breakdown of blood-retinal barrier and upregulation of cellular adhesion molecules. Invest Ophthalmol Vis Sci. (2003) 44:226–34. doi: 10.1167/iovs.01-1202 12506079

[18] Xu H Dawson R Crane IJ Liversidge J . Leukocyte diapedesis *in vivo* induces transient loss of tight junction protein at the blood-retina barrier. Invest Ophthalmol Vis Sci. (2005) 46:2487–94. doi: 10.1167/iovs.04-1333 15980240 PMC2478725

[19] Barros Ferreira L Ashander LM Ma Y Appukuttan B Williams KA Best G . Effects of tumor necrosis factor-α and interleukin-1β on human retinal endothelial cells. Cytokine. (2024) 173:156407. doi: 10.1016/j.cyto.2023.156407 37924741

[20] Zhang L Borjini N Lun Y Parab S Asonye G Singh R . CDCP1 regulates retinal pigmented epithelial barrier integrity for the development of experimental autoimmune uveitis. JCI Insight. (2022) 7:e157038. doi: 10.1172/jci.insight.157038 35951427 PMC9675461

[21] Quinn J Salman A Paluch C Jackson-Wood M McClements ME Luo J . Single-cell transcriptomic analysis of retinal immune regulation and blood-retinal barrier function during experimental autoimmune uveitis. Sci Rep. (2024) 14:20033. doi: 10.1038/s41598-024-68401-y 39198470 PMC11358488

[22] Feng Y Venema VJ Venema RC Tsai N Behzadian MA Caldwell RB . VEGF-induced permeability increase is mediated by caveolae. Invest Ophthalmol Vis Sci. (1999) 40:157–67. 9888439

[23] Galaris D Barbouti A Pantopoulos K . Iron homeostasis and oxidative stress: an intimate relationship. Biochim Biophys Acta Mol Cell Res. (2019) 1866:118535. doi: 10.1016/j.bbamcr.2019.118535 31446062

[24] Dixon SJ Patel DN Welsch M Skouta R Lee ED Hayano M . Pharmacological inhibition of cystine-glutamate exchange induces endoplasmic reticulum stress and ferroptosis. eLife. (2014) 3:e02523. doi: 10.7554/eLife.02523 24844246 PMC4054777

[25] Park E Chung SW . ROS-mediated autophagy increases intracellular iron levels and ferroptosis by ferritin and transferrin receptor regulation. Cell Death Dis. (2019) 10:822. doi: 10.1038/s41419-019-2064-5 31659150 PMC6817894

[26] Doll S Proneth B Tyurina YY Panzilius E Kobayashi S Ingold I . ACSL4 dictates ferroptosis sensitivity by shaping cellular lipid composition. Nat Chem Biol. (2017) 13:91–8. doi: 10.1038/nchembio.2239 27842070 PMC5610546

[27] Sun Y Zheng Y Wang C Liu Y . Glutathione depletion induces ferroptosis, autophagy, and premature cell senescence in retinal pigment epithelial cells. Cell Death Dis. (2018) 9:753. doi: 10.1038/s41419-018-0794-4 29988039 PMC6037763

[28] Wei TT Zhang MY Zheng XH Xie TH Wang W Zou J . Interferon-γ induces retinal pigment epithelial cell ferroptosis by a JAK1–2/STAT1/SLC7A11 signaling pathway in age-related macular degeneration. FEBS J. (2022) 289:1968–83. doi: 10.1111/febs.16272 34741776

[29] Gu Y Hao S Liu KY Gao M Lu B Sheng F . Airborne fine particulate matter (PM_2.5_) damages the inner blood-retinal barrier by inducing inflammation and ferroptosis in retinal vascular endothelial cells. Sci Total Environ. (2022) 838:156563. doi: 10.1016/j.scitotenv.2022.156563 35690207

[30] Li Y Wen Y Liu X Li Z Lin B Deng C . Single-cell RNA sequencing reveals a landscape and targeted treatment of ferroptosis in retinal ischemia/reperfusion injury. J Neuroinflamm. (2022) 19:261. doi: 10.1186/s12974-022-02621-9 36289494 PMC9597965

[31] Yao F Peng J Zhang E Ji D Gao Z Tang Y . Pathologically high intraocular pressure disturbs normal iron homeostasis and leads to retinal ganglion cell ferroptosis in glaucoma. Cell Death Differ. (2023) 30:69–81. doi: 10.1038/s41418-022-01046-4 35933500 PMC9883496

[32] Chen Y Fang ZM Yi X Wei X Jiang DS . The interaction between ferroptosis and inflammatory signaling pathways. Cell Death Dis. (2023) 14:205. doi: 10.1038/s41419-023-05716-0 36944609 PMC10030804

[33] He S Yaung J Kim YH Barron E Ryan SJ Hinton DR . Endoplasmic reticulum stress induced by oxidative stress in retinal pigment epithelial cells. Graefes Arch Clin Exp Ophthalmol. (2008) 246:677–83. doi: 10.1007/s00417-008-0770-2 18278507

[34] Lee YS Lee DH Choudry HA Bartlett DL Lee YJ . Ferroptosis-induced endoplasmic reticulum stress: cross-talk between ferroptosis and apoptosis. Mol Cancer Res. (2018) 16:1073–6. doi: 10.1158/1541-7786.MCR-18-0055 29592897 PMC6030493

[35] Levine B Mizushima N Virgin HW . Autophagy in immunity and inflammation. Nature. (2011) 469:323–35. doi: 10.1038/nature09782 21248839 PMC3131688

[36] Santeford A Wiley LA Park S Bamba S Nakamura R Gdoura A . Impaired autophagy in macrophages promotes inflammatory eye disease. Autophagy. (2016) 12:1876–85. doi: 10.1080/15548627.2016.1207857 27463423 PMC5066937

[37] Wang C Zhou W Su G Hu J Yang P . Progranulin suppressed autoimmune uveitis and autoimmune neuroinflammation by inhibiting Th1/Th17 cells and promoting Treg cells and M2 macrophages. Neurol Neuroimmunol Neuroinflamm. (2022) 9:e1133. doi: 10.1212/NXI.0000000000001133 35082168 PMC8791655

[38] Liu J Liao X Li N Xu Z Yang W Zhou H . Single-cell RNA sequencing reveals inflammatory retinal microglia in experimental autoimmune uveitis. MedComm. (2024) 5:e534. doi: 10.1002/mco2.534 38585235 PMC10999176

[39] Efimova I Catanzaro E Van der Meeren L Turubanova VD Hammad H Mishchenko TA . Vaccination with early ferroptotic cancer cells induces efficient antitumor immunity. J Immunother Cancer. (2020) 8:e001369. doi: 10.1136/jitc-2020-001369 33188036 PMC7668384

[40] Wiernicki B Maschalidi S Pinney J Adjemian S Vanden Berghe T Ravichandran KS . Cancer cells dying from ferroptosis impede dendritic cell-mediated anti-tumor immunity. Nat Commun. (2022) 13:3676. doi: 10.1038/s41467-022-31218-2 35760796 PMC9237053

[41] Luo X Gong HB Gao HY Wu YP Sun WY Li ZQ . Oxygenated phosphatidylethanolamine navigates phagocytosis of ferroptotic cells by interacting with TLR2. Cell Death Differ. (2021) 28:1971–89. doi: 10.1038/s41418-020-00719-2 33432112 PMC8185102

[42] Mbah NE Sutton D Hong HS Singhal R Menjivar RE Perricone MD . Tumor cell death by ferroptosis contributes to an immunosuppressive tumor microenvironment in syngeneic murine models of cancer. Cancer Metab. (2026) 14:9. doi: 10.1186/s40170-026-00428-3 41935249 PMC13072669

[43] Davidson AJ Hussey R Das J Overholtzer M Wood W . Ferroptosis-like cell death promotes and prolongs inflammation in Drosophila. Nat Cell Biol. (2024) 26:1535–44. doi: 10.1038/s41556-024-01450-7 38918597 PMC11392819

